# Reducing risks of fetal injury and stillbirths caused by infection/inflammation using healthy behaviors

**DOI:** 10.1186/1471-2393-15-S1-A10

**Published:** 2015-04-15

**Authors:** James A McGregor, Janice I French, Jim Christian, Marti Perhach, Josh Jones

**Affiliations:** 1 Dept. of Pediatrics, University of Colorado, Denver, Colorado, USA; 2 LA Best Babies Network, Los Angeles, California, USA; 3 PHCC, LP, Pueblo, Colorado, USA; 4 Group B Strep International, Pomona, California, USA

## Background

Potentially preventable morbid or lethal vertical infections are more common in pregnancy than is recognized [1]. Research suggests about 11% of stillbirths (SBs) in developed countries are caused by infection versus WHO-sponsored estimates of 38% worldwide [2,3]. Advances in diagnostic technologies, pregnancy immunology, and systematic surveys (“Human Microbiome Project”) have enabled new understanding of primary prevention of pregnancy/lactation-associated infection [3,4]. What is lacking is a systematic vigorously designed and adequately funded research agenda to provably reduce risks of individual or population-based risks of pregnancy infection. Lacking such “evidence-based” recommendations, some researchers suggest that, except for syphilis and vaccine-preventable infections, there are no satisfactory proven approaches to prevent infection-caused stillbirth [3]. Therefore, we used accumulated knowledge to formulate behavioral “no/low cost” and practicable/actionable pathobiologically and behaviorally informed recommendations to allow families and policy makers to reasonably reduce risks of maternal and pregnancy infection that cause SB. Evidence-based recommendations await controlled trials in suitable populations.

Changes in personal (“lifestyle”) behaviors are now demonstrated to be cost-effective means to enhance individual and population measures of complex chronic diseases. The Institute of Medicine strongly recommends behavioral approaches for preventing common complex diseases such as coronary artery disease (CAD) and stroke [5].

**Figure 1:**
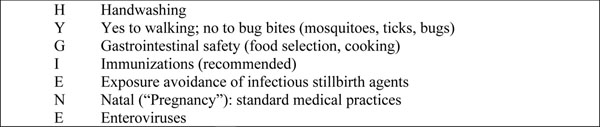
“HYGIENE” as a students’ mnemonic for healthy behaviors to reduce risk of infection-caused stillbirth or fetal injury

Using short slogans, such as “safe sex”, or acronyms, such as “DASH”, as well as providing mnemonic prompts, can be helpful for remembering to change personal behaviors. In this paper, we propose the mnemonic, “HYGIENE”, to assist in promoting safe pregnancy behaviors to reduce risks of common infections associated with stillbirth (Figure 1). (“HYGIENE” also denotes the Greek mythologic goddess of health and healthy behaviors.) Some of these behaviors are listed with the associated potentially preventable illness/agent in Table 1. This list is not meant to be comprehensive, but identifies “high impact pathogens” commonly listed as causing fetal death. Importantly, the commonest is malaria and the most lethal is the hemorrhagic Ebola virus infection.

**Table 1:** Primary behaviors for prevention of infection-caused fetal injury or death (stillbirth)

Behavior	Agents/Illness
1. “Safe food” (selection, preparation, handwashing)	*Listeria monocytogenes* Enteropathogens (*E. coli, Salmonella ssp*.) *Toxoplasma gondii* Enteroviruses Ebola (per the CDC, Ebola is not spread in general by food; however, in Africa, Ebola may be spread as a result of handling bushmeat)

2. “Safe sex” (no new partners)	HSV (herpes) 1 and 2 STIs, HIV Syphilis Chlamydia Gonorrhea CMV Ebola

3. “No (bug) bites” (zoonosis, mosquitoes, ticks, flies) and “avoid exposure to infectious animals”	Malaria Malaria-like infections Dengue West Nile Virus Tickborne infections (Rocky Mountain spotted fever, etc.) Q fever Lymphocytic choriomeningitis virus (LCMV) Leptospirosis Ebola

4. “Hygiene and oral health” (reduce body fluid exposure and bad mouth bacteria/inflammation)	CMV HSV 1-6 Hepatitis A, B, C Periodontal microorganisms Ebola

5. “Pregnancy” (follow CDC-recommended protocols)	Group B *Streptococcus* (GBS) Influenza

6. “Optimize pregnancy and birth management to reduce/eliminate ascending infections”	Vaginal/cervical infections GBS protocols Chorioamnionitis Transfusions

“H” prompts the personal imperatives of handwashing to reduce risks of multiple infections (listeriosis, toxigenic *E. coli*, and many enteropathogens as well as hand-to-hand spread of influenza viruses) acquired by fecal handling. Handwashing is strongly suggested (without formal evidence) for prevention of cytomegalovirus (CMV) infection during pregnancy, especially among medical personnel and caretakers of toddlers [6].

“Y” prompts the slogan’s “yes” to walking and exercise, but “no” to insect bites including both 1) mosquitoes which can cause malaria, malaria-like parasitemias, dengue fever agents, West Nile virus and other viral encephalopathies, and 2) multiple tick vectors for Rocky Mountain spotted fever and Lyme disease (*Borrelia burgdorferi*). The CDC widely recommends means to avoid tick bites (such as avoiding tick-infested areas), routine examination for ticks, and prompt, safe removal if found [7]. Means to prevent mosquito bites include elimination of possible breeding areas and mosquito bed netting in malarial areas. [8].

“G” prompts prevention of gastrointestinal illness, including reducing risks of listeriosis (*Listeria monocytogenes*) and toxoplasmosis (*Toxoplasma gondii*) [9,10] as well as enteropathogens, not only by handwashing, but also by safe food selection, preparation, and handling [11].

“I” prompts performance of CDC-recommended immunizations, including rubella, tetanus, influenza, pertussis, viral hepatitis, and yellow fever, in travellers to endemic areas. Newer vaccines such as the tetrapotent vaccine for dengue fever are proven effective, but not yet recommended in pregnancy. Vaccines against multiple other stillbirth-causing infections, including herpes viruses (HSV 1 and 2), cytomegalovirus, other microorganisms including group B *Streptococcus*, *Leptospira*, the agents of Q fever and malaria, and common sexually transmitted infections as well as Ebola and human parvovirus (HPV-B19), may hold considerable promise if they become available [12,13].

“E” mandates consideration of avoiding exposure to infectious stillbirth agents including CMV and human parvovirus (HPV-B19) among susceptible women (daycare providers, teachers, medical personnel, and others who care for children with potentially infectious secretions and coughs). Other infections potentially preventable by eliminating exposure include malaria, malaria-like infections, Lyme disease, and multiple other mosquito and tickborne vector-transmitted infections. Importantly, meth mothers and their sexual partners can prevent infections including syphilis and other sexually transmitted infections/diseases (STIs/STDs) and HIV by avoiding unsafe sexual practices [14].

“N” stands for “natal” (“pregnancy”) and prompts recognition of pregnancy providers’ “standard of practice” responsibilities to complete recommended screening and indicated treatment of stillbirth-implicated infections including syphilis, rubella, urinary tract infections and bacteriuria, and abnormal vaginal microflora including bacterial vaginosis (BV) and group B *Streptococcal* infection or colonization [15].

New expert clinical suggestions for early pregnancy GBS screening by routine antenatal urine culture are increasingly voiced. Some experienced clinicians recommend routine GBS bacteriuria testing for every pregnant woman and, if positive, treatment with oral penicillin. Treatment for any infection in pregnancy is to be followed by a confirmatory “test of cure”. Other obstetrical checklist items are listed in Table 1. New clinical recommendations to prevent “ascending” intrauterine infection include optimizing labor care to prevent “dystocia” and vigorous screening and treatment of all abnormal vaginal bacteria “dysbiosis”. Intrusive “stripping of membranes” to induce labor is both clinically ineffective and may transmit potential cervico-vaginal pathogenic microorganisms into the uterus. Avoidance of this practice is recommended by some experts.

Finally “E” reminds families and practitioners of the increasing role of microbes’ ability to induce damaging placental and fetal inflammation. Work by Nuova and others have shown that multiple types of microorganisms that cause placental inflammation, including enteroviruses (especially Coxsackie viruses), are increasingly implicated in both abortion and stillbirth [16].

Except for syphilis and both influenza and viral hepatitis which are vaccine-preventable, neither enteroviruses or the more common clinically recognizable infection causes of fetal death have reliably proven primary prevention strategies. In the absence of proven vaccination practices, “HYGIENE”-prompted “healthy pregnancy behaviors“ by both families and pregnancy providers offer potentially powerful protection against stillbirth-associated infections until more specific prevention strategies including vaccination are demonstrated in well-controlled trials and authoritatively recommended.
